# pTcINDEX: a stable tetracycline-regulated expression vector for *Trypanosoma cruzi*

**DOI:** 10.1186/1472-6750-6-32

**Published:** 2006-07-06

**Authors:** Martin C Taylor, John M Kelly

**Affiliations:** 1 London School of Hygiene and Tropical Medicine, Keppel Street, London WC1E 7HT, UK

## Abstract

**Background:**

*Trypanosoma cruzi *is a protozoan pathogen of major medical importance in Latin America. It is also an early diverging eukaryote that displays many unusual biochemical features. The completion of the *T. cruzi *genome project has highlighted the need to extend the range of techniques available to study gene function. To this end we report the development of a stable tetracycline-dependent expression vector applicable to this parasite and describe in detail the parameters of the system.

**Results:**

We first produced *T. cruzi *cell lines that constitutively expressed bacteriophage T7 RNA polymerase and the tetracycline repressor protein from a multicopy episome. An integrative vector with an inducible expression site under the control of a tetracycline-regulatable T7 promoter (pTcINDEX) was targeted to the transcriptionally silent ribosomal RNA spacer region of these parasites and transformants selected using a T7 RNA polymerase-dependent hygromycin resistance gene. To test the system we used two marker proteins, luciferase and red fluorescent protein (RFP), and an endogenous parasite protein (a mitochondrial superoxide dismutase). In each case we found that induction was both time and dose-dependent. Luciferase mRNA could be induced by at least 100-fold, and luciferase activity up to 60-fold, within 24 hours of the addition of tetracycline. When we examined RFP induction by confocal microscopy and fluorescence activated cell sorter, we observed very high levels of expression (>1000-fold increase in fluorescence intensity), although this was not synchronous throughout clonal populations. Induction of superoxide dismutase resulted in an 18-fold increase in cellular activity. The observation that a tagged version of the enzyme was correctly targeted to the mitochondrion demonstrates that our expression system may also provide a high-throughput strategy for subcellular localisation.

**Conclusion:**

Our results show that pTcINDEX represents a valuable addition to the genetic tools available for *T. cruzi*. The vector system is sufficiently flexible that it should have widespread uses including inducible expression of tagged proteins, generation of conditional knockout cell lines and the application of dominant-negative approaches.

## Background

*Trypanosoma cruzi*, the agent of Chagas disease, is a member of the Kinetoplastidae, an early-diverging group of protozoa. This organism is the most important parasite in Latin America, while its close relatives *Trypanosoma brucei *and *Leishmania *cause African sleeping sickness and the leishmaniases respectively. In addition to their medical and veterinary significance, trypanosomes have been studied as examples of primitive eukaryotes. They show several biological peculiarities which have made them subjects of great interest. These include polycistronic transcription, *trans*-splicing of mRNA, mitochondrial RNA editing, compartmentalisation of glycolysis and the utilisation of a unique thiol, trypanothione, in place of glutathione. Genome sequencing projects have recently been completed for each of the human pathogenic trypanosomatids, *T. cruzi, T. brucei *and *Leishmania *[1-3]. To fully exploit this vast amount of information it is essential that efforts are made to improve and extend the range of tools available for analysing the function of genes *in vivo*. This is particularly the case with *T*. *cruzi*, where technical limitations currently restrict analysis of biological function.

The last few years have seen an explosion of new data on gene function in *T. brucei*, largely due to the development of regulated systems that allow inducible expression of both protein and double-stranded RNA [4-9]. These systems can facilitate the study of gene function by over-expression [10], conditional knockout [11], or by RNA interference (RNAi)-mediated down-regulation of gene expression [8,9,11,12]. RNAi is currently the method of choice for the analysis of gene function in *T. brucei *and can be used to inform studies on *T. cruzi *and *Leishmania *genes which have orthologues in *T. brucei*. However many trypanosomatid genes are species-specific [13]. Since *T. cruzi *lacks the machinery for RNAi, specifically the *AGO1 *gene [14,15], our unpublished observations), approaches such as gene deletion or expression of dominant-negative mutant proteins are of critical importance for studying function. However, both gene knockout and expression of mutant proteins can produce a lethal or deleterious phenotype. It would therefore be advantageous to have a system that allows expression of transgenes in a controlled and repressible manner.

In general, trypanosomes do not appear to control expression of protein coding genes at the level of transcription initiation. The exceptions to this are the major surface glycoprotein genes of procyclic, metacyclic and bloodstream forms of *T. brucei *[16,17], where RNA polymerase I (pol I)-dependent promoters can drive expression in a developmental and locus specific manner. RNA polymerase II (pol II)-dependent promoters for protein coding genes have not been unequivocally identified in trypanosomatids and there are no known examples of inducible transcription units. Consequently, it has been necessary to import regulatable genetic machinery from other organisms to create artificial inducible expression systems. Such a system for *T. brucei *was first developed by Wirtz and Clayton [4]. This relies on the bacterial tetracycline repressor protein (tetR) to block transcription from an engineered promoter in the absence of tetracycline. On addition of tetracycline, the repressor is released from the DNA and transcription is allowed to proceed. Initially, use was made of the *T. brucei *procyclin promoter [4]. However, the system was found to be tightly regulated to a similar degree when a bacteriophage T7 promoter was utilised [7]. This necessitated the integration of a T7 RNA polymerase gene into a transcriptionally active region of the trypanosome genome prior to insertion of the construct containing the inducible gene. A similar regulatable expression system has now also been described for *Leishmania *based on an inducible copy of the endogenous ribosomal RNA (rRNA) promoter [18].

In *T. cruzi*, inducible expression following transient transfection with a plasmid has been reported [19]. More recently a stable system has been reported by DaRocha et al. [15], in which the T7 polymerase and *tetR *genes were inserted into the tubulin gene array together with the strong rRNA promoter. The effects, if any, of this promoter on expression of endogenous genes flanking the insertion were not described, although a similar vector used in *T. brucei *caused upregulation of genes downstream of the integration site [20]. Detailed characterisation of this inducible cell line was not undertaken to assess the parameters of regulated expression. There have been no further reports on its use or applications.

Here we describe a stable tetracycline-inducible expression vector for *T. cruzi *that circumvents some of the potential problems associated with integration into an endogenously transcribed locus. The system is based on an integrative vector that facilitates inducible expression of specific genes in a transcriptionally quiescent locus and engineered cell lines that constitutively express the T7 RNA polymerase and *tetR *genes from an episomal background. These experiments now provide a framework for using stable inducible expression as a tool for studying gene function in *T. cruzi*.

## Results

### Production of cell lines stably expressing tetR and T7 RNA polymerase

**Figure 1:**
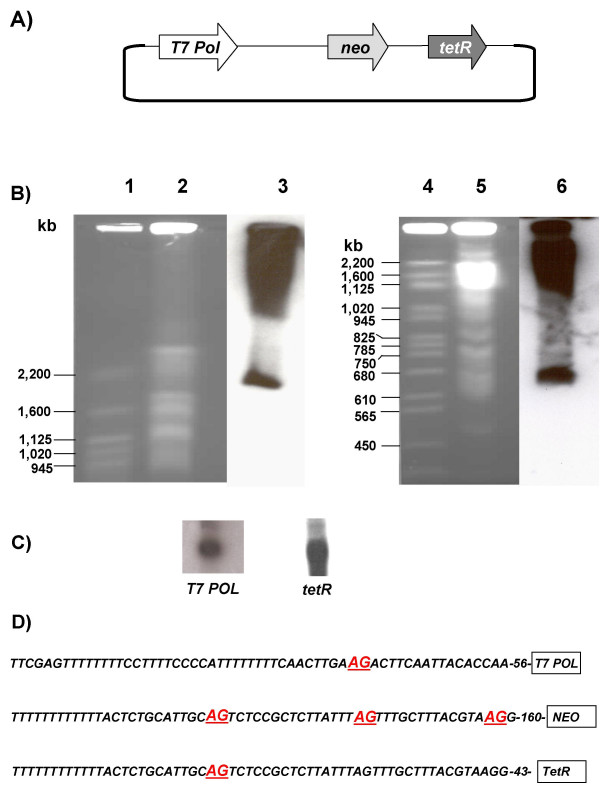
**Production of cell lines stably expressing tetR and T7 RNA polymerase**. (A) Simplified map of pLEW13 indicating the relative locations of the three transgenes [7]. (B) CHEFE analysis of chromosomal DNA isolated from CL-Brener [pLEW13] epimastigotes showing aberrant migration of the pLEW13 DNA. Lanes 1–3, a 0.8% PFC agarose CHEFE gel (auto-algorithm set for 300 kb-3 Mb separation). Lanes 1 (*Saccharomyces cerevisiae *size standards (Bio-Rad)) and 2 (CL-Brener [pLEW13]), the ethidium bromide stained gel. Lane 3, an autoradiograph obtained with the T7 RNA polymerase probe. Lanes 4–6, a 1.0% PFC agarose CHEFE gel (auto-algorithm set for 300 kb-1 Mb). Lanes 4 (*S. cerevisiae *size standards) and 5 (CL-Brener [pLEW13]), the ethidium bromide stained gel. Lane 6 is an autoradiograph obtained with the T7 RNA polymerase probe. Molecular sizes are given in kb. (C) Expression of the transgenes for T7 RNA polymerase and *tetR *in pLEW13 transformed epimastigotes. 10 μg total RNA was blotted and hybridised with either the T7 RNA polymerase (*T7 POL*) or *tetR *probes. (D) Splice acceptor sites used by *T. cruzi *to process the transcripts as mapped by RT-PCR. The AG dinucleotide sites of spliced leader addition identified following sequencing of the RT-PCR products are red and underlined. The numbers adjacent to the boxes indicate the distance in nucleotides between the sequence shown and the start codon of each gene. The T7 RNA polymerase is flanked by the *T. brucei *procyclin spliced leader acceptor site, whereas both *neo *and *tetR *are flanked by *T. brucei *actin spliced leader acceptor sites. In the case of the T7 RNA polymerase and *tetR *transcripts, only one addition site was identified; in the case of the *neo *transcript, three were found.

Plasmid pLEW13, a construct designed to target the *T. brucei *β-tubulin locus, contains both T7 RNA polymerase and *tetR *genes with *neo *as a selectable marker [7] (Fig. 1A). We electroporated *T. cruzi *CL-Brener epimastigotes with circular pLEW13 DNA (a gift from George Cross) and selected recombinant parasites on 200 μg ml^-1 ^G418. Stably transformed parasites were obtained after six weeks, even though this vector contains no *T. cruzi*-derived sequences. Southern analysis showed that the transformants contained multiple copies of the input construct organised in a tandem array (data not shown). Circular DNA in transformed *T. cruzi *usually replicates as an episome of head-to-tail repeats of the input construct [21]. However, since the vector contained *T. brucei *β-tubulin coding sequences, which are very similar to the corresponding *T. cruzi *gene (88% overall nucleotide identity, up to 96% in some regions), it was important to establish whether the pLEW13 tandem array was a circular episome or had resulted from multiple integrations into the tubulin locus.

Circular molecules show aberrant migration on pulsed field gels as their movement is independent of their molecular mass, in contrast to linear chromosomes. DNA from CL-Brener epimastigotes transformed with pLEW13 (CL-Brener [pLEW13]) was therefore subjected to contour-clamped homogenous electric field gel electrophoresis analysis (CHEFE) under differing separation conditions (Fig. 1B). Using parameters designed to separate the larger molecules (up to 3 Mb), the T7 RNA polymerase probe hybridised to a band of approximately 2 Mb and to a smear of higher molecular weight material (Fig. 1B lane 3). When the DNA was fractionated under conditions optimal for separation of molecules of between 300 kb and 1 Mb, the hybridising band ran at 680 kb, again accompanied by a smear of apparently higher molecular weight material (Fig. 1B lane 6). The migration of the pLEW13 construct is therefore independent of its molecular weight indicative of a circular episome containing multiple copies of the T7 RNA polymerase and *tetR *genes. Southern analysis of genomic DNA also indicated no linkage between the *T. cruzi *α-tubulin genes and the T7 RNA polymerase (data not shown).

To check expression of the transfected genes, RNA was prepared and analysed by northern blotting. This showed that both T7 RNA polymerase and the *tetR *gene were expressed at high levels (Fig. 1C). In trypanosomes each mRNA is processed by *trans*-splicing which results in the addition of a 5' spliced leader sequence of 39 nucleotides [22]. Since the RNA processing signals in pLEW13 were derived from *T. brucei *it was necessary to establish that the transgenic mRNAs were correctly spliced. For each gene, primers were designed to sequences approximately 150–250 bp into the ORF and used in conjunction with a primer to the *T. cruzi *spliced leader in an RT-PCR reaction (Methods). The resulting products were cloned and sequenced. Each splice addition site could be mapped to an AG dinucleotide located downstream of a polypyrimidine tract (Fig. 1D). In the case of the *T. brucei *actin intergenic sequence upstream of *neo*, three separate splice sites were identified, all of which were upstream of the start codon. Only one of these was identified in the *tetR *mRNA which has the same flanking sequence. In the case of the procyclin splice acceptor site upstream of the T7 RNA polymerase, only the site previously mapped in *T. brucei *was utilised [23]. This analysis indicated that the *T. brucei *RNA processing signals were being correctly utilised by *T. cruzi *and that the T7 RNA polymerase and *tetR *mRNAs were therefore likely to be functional.

### Features of the tetracycline inducible expression vector

**Figure 2:**
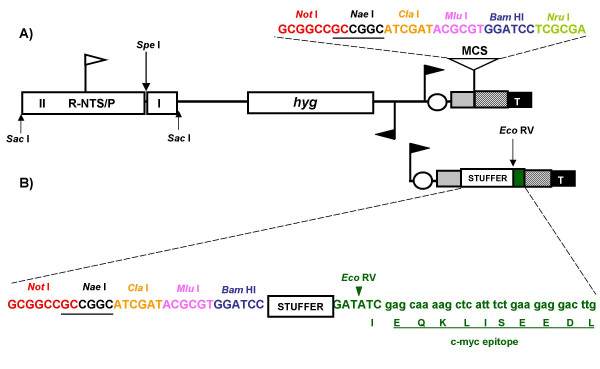
**The *T. cruzi *inducible expression vectors pTcINDEX and pTcINDEX-C-myc**. (A) Map of pTcINDEX. The grey box adjacent to the multiple cloning site (MCS) indicates the ribosomal protein P2β splice acceptor site [25]. The hatched box indicates the *T. cruzi *actin intergenic region. The black box (T) is the T7 transcriptional terminator. Black flags represent T7 promoters and the oval identifies the location of the tet operator. R-NTS/P is the ribosomal non-transcribed spacer and promoter region used to target the construct. Roman numerals I and II indicate the two halves of the targeting sequence which are cloned in the opposite order to their position in the genome (see Fig. 3A). The white flag indicates the location of the pol I transcription start site [24]. Following insertion of a gene of interest into the MCS, the construct can be linearised with *Spe *I (dotted line) to facilitate targeting to the rRNA non-transcribed spacer region. The vector is built on a pUC19 backbone (not illustrated for clarity) and confers ampicillin resistance on *E. coli*. The sequence of the MCS is shown above the map indicating useful restriction sites. *Nae *I and *Nru *I were incorporated as blunt end sites to facilitate cloning of genes which contain the other restriction sites. (B) Map of pTcINDEX-C-myc. The inducible cassette alone is shown for clarity. The rest of the vector is identical to pTcINDEX. Features are as shown in A, except that the *BPP1-myc *fusion gene has been inserted into the *Bam *HI/*Nru *I sites of pTcINDEX (Methods). The white box labelled "stuffer" indicates the dispensable BPP1 ORF [48]. This can be removed by digestion with one of the MCS enzymes and *Eco *RV and replaced with the gene of interest. The c-myc epitope tag is indicated by a green box. The *Eco *RV cleavage site and the translated sequence of the c-myc tag (underlined) are indicated to allow easy design of in-frame fusions with the epitope tag. Note the *Nru *I site is absent in this plasmid.

The inducible expression vector pTcINDEX (Fig. 2A) was designed to integrate into the non-transcribed ribosomal RNA spacer region upstream of the pol I-mediated transcription start site [24] (Methods). We targeted this region because, to our knowledge, it is the only section of the *T. cruzi *genome so far identified as being transcriptionally silent. In addition, the level of sequence conservation at this locus suggested that the construct could be targeted to the corresponding region in multiple parasite strains. The targeting fragment is cloned as a *Sac *I cassette which can be readily replaced to allow integration elsewhere in the genome.

As a drug selectable marker we used the hygromycin B phosphotransferase (*hyg*) gene under the control of a non-repressible T7 promoter, thus converting antibiotic resistance into a digenic trait. In pLEW13 transformed cells that constitutively express the T7 RNA polymerase, this arrangement serves a second function. In the presence of hygromycin, the requirement for T7 RNA polymerase to drive expression of *hyg *removes the necessity for the continued use of G418 to maintain the pLEW13 construct and selects for trypanosomes with active T7 RNA polymerase. The inducible expression cassette in the pTcINDEX vector contains a tetracycline-dependent T7 promoter, with the tet operator sequence (tetO, cCTATCAgTGATAGa, where upper case indicates bases important in tetR binding) placed immediately downstream. The multiple cloning site is flanked at its 3'-end by the intergenic sequence from the *T. cruzi *actin locus to provide a polyadenylation signal, and at the 5'-end by the splice acceptor site from the ribosomal protein P2β locus. Sequences from this region have been shown to enhance the expression of transfected genes [25]. Finally, we incorporated a T7 RNA polymerase transcription terminator into the construct to block run-through transcription of sequences downstream of the integration site.

**Figure 3:**
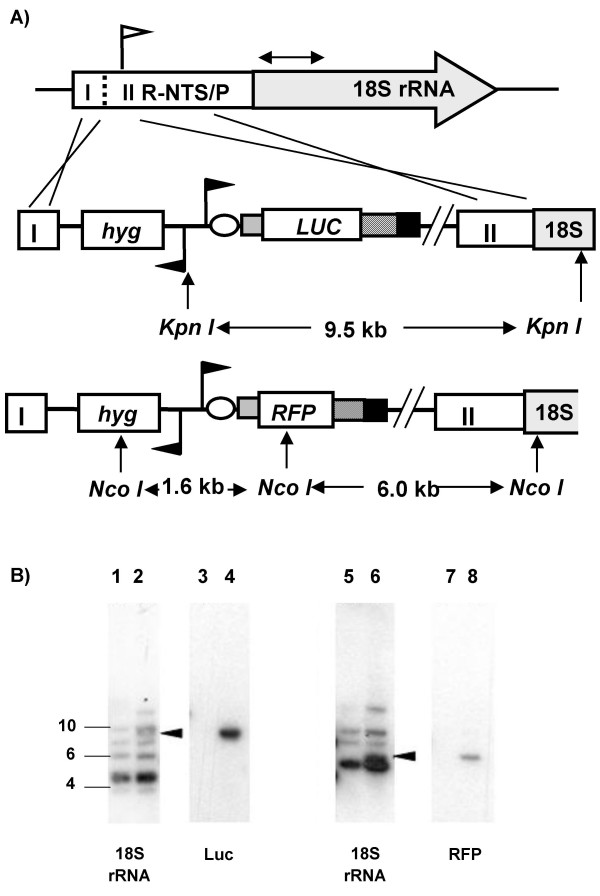
**Integration of pTcINDEX-Luc and pTcINDEX-RFP into the ribosomal non- transcribed spacer**. (A) Configuration of correctly targeted constructs showing relevant restriction sites. R-NTS/P represents the ribosomal non-transcribed spacer/promoter region with the white flag indicating the promoter [24]. The targeting fragment is designed to integrate upstream of the rRNA transcription start. The dotted line represents the position of the *Spe *I site introduced into the spacer to facilitate linearization. This site is absent from the genomic DNA. The crossed lines indicate the sites of homologous recombination. The double headed arrow shows the region of the 18S rRNA gene used as a probe when assessing integration. The other symbols are as in Fig. 2. The configurations for integration of pTcINDEX-Luc and pTcINDEX-RFP are shown. The expected fragment size following a targeted integration is illustrated below each map. (B) Southern analysis of the pTcINDEX-Luc and pTcINDEX-RFP transformants Arrowheads indicate fragments specific to the transformants following hybridisation with the 18S rRNA probe. These bands also hybridise specifically to the full-length luciferase or RFP probes. Lanes 1,3,5,7 contain DNA from CL-Brener [pLEW13], lanes 2 and 4 from CL-Brener:pTcINDEX-Luc [pLEW13]. Lanes 6 and 8 contain DNA from CL-Brener:pTcINDEX-RFP [pLEW13]. DNA in lanes 1–4 was digested with *Kpn *I and in lanes 5–8 with *Nco *I. The probes used are indicated below each autoradiograph. A second smaller band (1.6 kb) which hybridises to the 5' end of the RFP probe (lane 8) migrated off the bottom of this gel. Molecular sizes are shown in kb.

To test the capability of the vector to mediate tetracycline-regulatable expression we cloned the genes encoding firefly luciferase (Luc) and red fluorescent protein (RFP) into the multiple cloning site (Fig. 2, Methods). *Spe *I linearised forms of the resulting constructs (pTcINDEX-Luc and pTcINDEX-RFP) were then used to transform CL-Brener [pLEW13] epimastigotes that constitutively express T7 RNA polymerase and tetR. Integration into the rRNA locus (illustrated in Fig. 3A) was confirmed by Southern analysis. This showed linkage of both of the transgenes to the endogenous 18S rRNA gene, (for examples, see Fig. 3). The appearance of novel fragments in the lanes containing DNA from the transformants (9.5 kb with the luciferase and 18S rRNA probes (Fig. 3B, lanes 2 and 4), 6 kb with the 18S rRNA and RFP probes (Fig. 3B, lanes 6 and 8)), which were absent from the CL-Brener [pLEW13] lanes, were diagnostic of targeted integration into the non-transcribed spacer region upstream of the 18S rRNA gene.

### Induction of luciferase in pTcINDEX-Luc transformants

**Figure 4:**
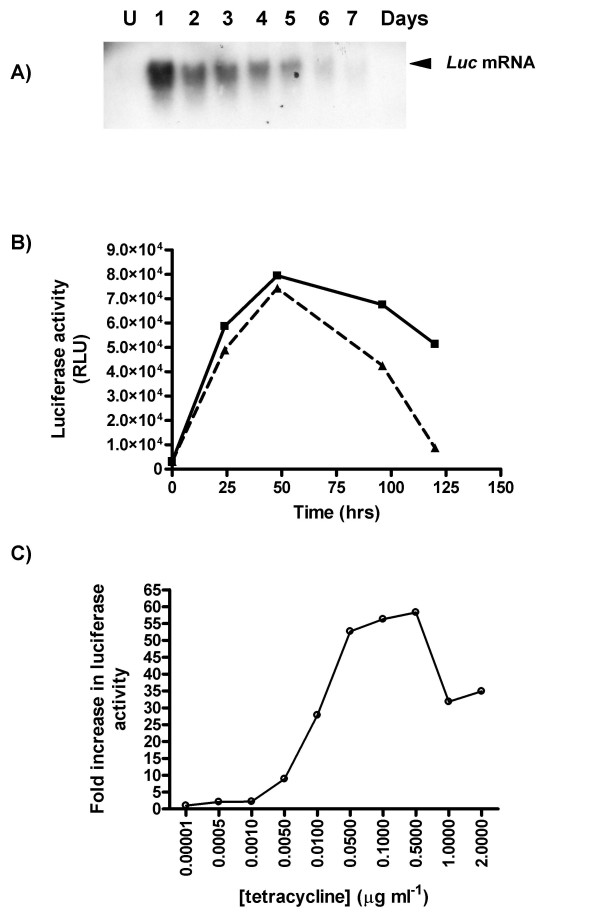
**Induction of luciferase by tetracycline**. (A) Expression of luciferase mRNA in a polyclonal line of tetracycline treated cells. pTcINDEX-Luc transformed epimastigotes were harvested each day following the addition of tetracycline to the cultures (5 μg ml^-1^). Lane U is the uninduced cell line. Lanes 1–7 represent days following induction. 1.25 μg total RNA was loaded in each lane. The blot was hybridised with a 1.7 kb *Bam *HI/*Sal *I fragment containing the luciferase ORF. (B) Luciferase activity over a time course. Cells were induced and aliquots removed for enzyme assay at indicated timepoints. Dashed line: cells were induced for 24 hours then washed twice in tetracycline-free medium and resuspended in tetracycline-free medium. Solid line: cells were induced with tetracycline then incubated with no further treatment. (C) Effect of tetracycline concentration on the level of luciferase activity. 10 identical flasks of epimastigotes (as above) were treated with different concentrations of tetracycline for 24 hours. Cells were harvested and the luciferase activity measured (Methods). The Y axis indicates the fold increase in luciferase activity above uninduced cells, normalised, to the amount of protein present in each extract.

We first investigated the induction of luciferase RNA in a polyclonal line of pTcINDEX-Luc transformed cells. Tetracycline was added once to epimastigotes in early mid-logarithmic growth phase (approximately 10^6 ^parasites ml^-1^) and aliquots removed every 24 hours for RNA purification. No further tetracycline was added during this period, as we wished to see if the gene returned to a repressed state. Northern analysis was performed (Fig. 4A) and the relative level of luciferase RNA measured at each time point using a phosphorimager. In the lane containing RNA from non-treated cells, the signal detected was not significantly above the background measured from an irrelevant piece of the membrane. This indicates a tightly regulated system with a very low level of "leaky" transcription. 24 hours after the addition of tetracycline, the level of luciferase mRNA was found to have increased dramatically (Fig. 4A). The mRNA levels at later time points declined gradually. The change in luciferase RNA levels was mirrored in the level of luciferase activity (Fig. 4B). The enzyme level increased considerably over 24 hours and continued to increase up to 48 hours. Thereafter it declined gradually. When the cells were washed after 24 hours exposure to tetracycline and resuspended in tetracycline-free medium, the luciferase activity reached a peak at 48 hours but had declined almost to background levels three days later (Fig 4B, dashed line).

To examine the relationship between tetracycline concentration and the induction of luciferase activity, a culture of pTcINDEX-Luc transformed CL-Brener [pLEW13] epimastigotes was divided into 10 individual flasks and tetracycline added at a range of concentrations (Fig. 4C). The cells were incubated for 24 hours, then harvested and the luciferase activity measured (Methods). There was negligible increase in luciferase activity over the level in non-treated cells at concentrations up to and including 1 ng ml^-1^. The increased activity became significant following treatment with 5 ng ml^-1 ^and continued to increase with concentration before levelling off at 500 ng ml^-1 ^(Fig. 4C). At tetracycline concentrations of 1 or 2 μg ml^-1^, the extent of induction decreased approximately two-fold, an effect that was reproducible. At higher levels of tetracycline there were detectable increases in parasite doubling time. The optimal increase of luciferase activity that was achieved was approximately 60-fold over background. This is less than the increased level of the corresponding transcript (Fig. 4A) and may indicate the presence of control mechanisms at the level of translation or instability of the luciferase protein in this context.

It has previously been noted that different clones transformed with the same tetracycline-regulated construct in *T. brucei *will exhibit differences in both the background level and the extent of inducible expression of the transfected gene [7,9,11]. This variability has been ascribed, in part, to epigenetic factors operating differentially on each site of integration [26]. To examine whether this variation occurred in *T. cruzi*, we isolated several clones from independent transfections. Variability was indeed observed (Table 1). The background level of luciferase activity varied from 700 to 5000 relative light units (RLU) per 5 × 10^4 ^cells. The background remained constant in a given clone over time. The level of induction after 24 hours varied from 2 to 37-fold between different clones in this experiment. In *T. cruzi *all the ribosomal RNA arrays are present at one chromosomal locus, in contrast to the situation in *T. brucei*. However the sequence across this locus is unavailable and it has not yet been possible to determine if there is a relationship between the level of expression and the site of integration.

### Cell-by-cell examination of the induction process using RFP

**Figure 5:**
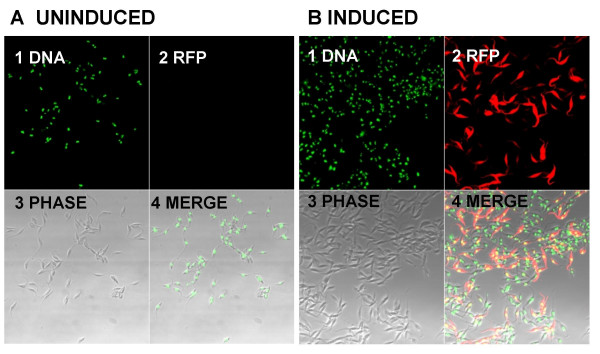
**RFP expression in cloned cells by microscopy**. Fluorescence microscopy of cloned pTcINDEX-RFP transformed epimastigotes before and after induction with tetracycline (5 μg ml^-1^). An aliquot was removed, and fixed every 24 hours for eight days. Cells were examined on a Zeiss LSM 510 microscope. Panel A shows the uninduced population and panel B the same culture 8 days after induction. In each case 1: DNA stained with TOTO-3 (green), 2: RFP expression (red), 3: phase image and 4 is a merged image.

To determine how individual cells responded to tetracycline, we examined clones isolated following transformation with pTcINDEX-RFP (Methods). Epimastigotes of clone CL:RFP C2 were maintained in tetracycline-free medium, then an aliquot was fixed onto a slide. Tetracycline was added to the remainder of the culture. An aliquot of cells was removed and fixed onto a slide every 24 hours for eight days. It was apparent that RFP expression had been highly induced by the third day, since the cell pellet had a red tinge visible to the eye. The cells were stained for DNA and examined by confocal microscopy. No red fluorescence was visible in the uninduced population (Fig. 5A). After 24 hours a few cells displayed faint fluorescence, and after three days some cells were extremely bright. The number of visibly fluorescent cells increased over time. After eight days the majority of cells exhibited some red fluorescence (Fig. 5B), although there was variation in the level. This suggested that induction, as measured using this parameter, does not occur at the same rate or to the same degree in all cells of a given clonal population.

**Figure 6:**
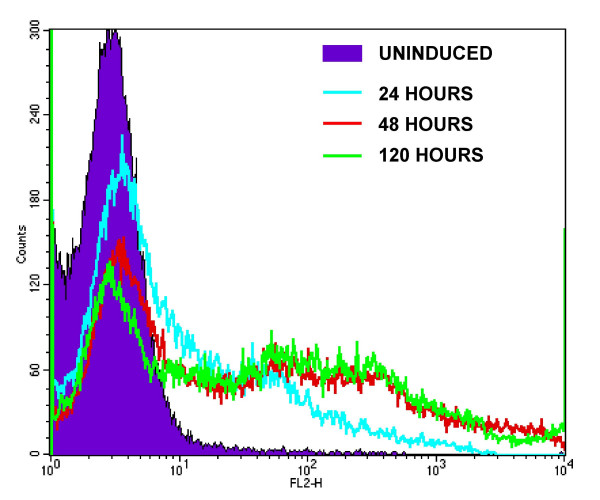
**FACS analysis of expression of RFP in a cloned cell line**. Tetracycline (0.5 μg ml^-1^) was added and samples were removed at specific timepoints. The level of fluorescence in the population was measured by counting 5 × 10^3 ^cells per timepoint on a FACScan. The filled curve is the uninduced population. The traces for induced cells are shown as overlaid lines: blue, 24 hours: red, 48 hours and green, 120 hours post-induction. The Y axis indicates the number of cells counted, whilst the X axis shows the level of RFP expression in AFU.

To examine this variation further we quantified the distribution of induced fluorescence in the population by FACS analysis (Fig. 6). In this experiment the tetracycline treatment was carried out at 0.5 μg ml^-1^, as this concentration was optimal for expression, at least in the case of luciferase (Fig. 4C). FACS analysis showed that in the first 24 hours post-induction, there was a significant shift in the fluorescence profile, with 35% of cells showing a 10–1000 fold increase in intensity (Fig. 6, blue line). The profile shifted rightwards over time but did not sharpen, indicating that fluorescence intensity varied between individual cells, confirming the observation made by microscopy (Fig. 5). The maximal shift was seen on day 5 (Fig. 6, green line), when 14 % of the cells were found to exhibit a 1000–10000 fold increase in fluorescence. Even at this stage however, 34% of cells remained in the 0–10 arbitrary fluorescence units (AFU) range.

We also examined the extent of variation in both background and inducibility in the RFP expressing clones. Again we observed a range of values (Table 2). For example, with clone CL:RFP B1 there was only slight induction, whereas all the other clones showed significant levels. In this experiment, tetracycline was added every three days to maintain the level of induction.

### Addition of an rRNA promoter to pLEW13 results in higher background expression levels

**Figure 7:**
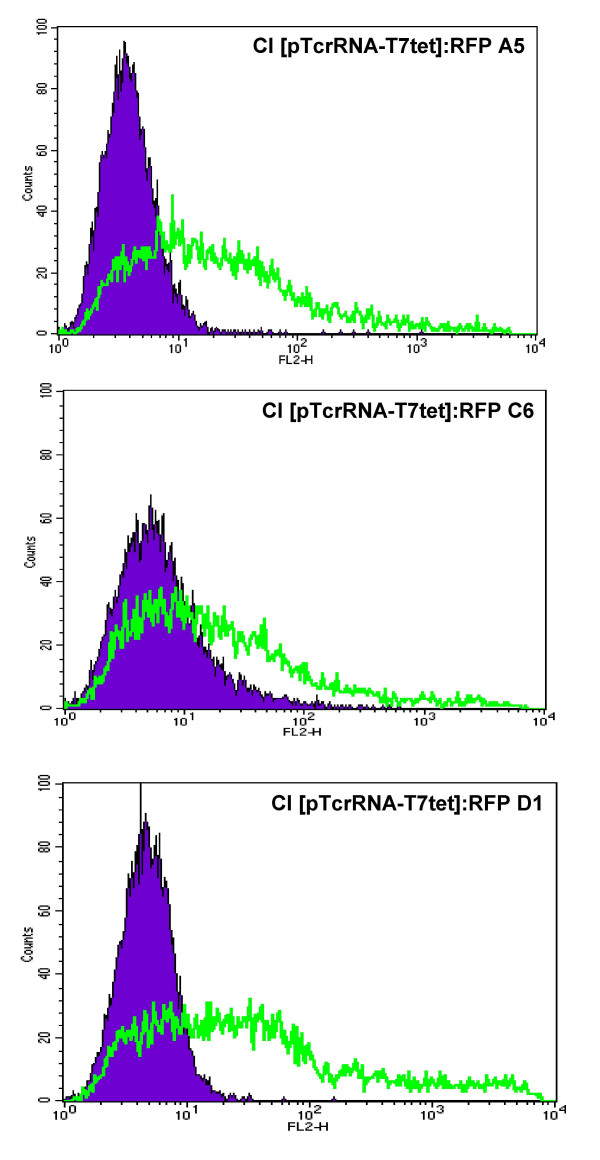
**FACscan analysis of expression of RFP in 3 independent clones in the CL [pTcrRNA-T7tetR] background**. Tetracycline (0.5 μg ml^-1^) was added and samples were removed and fixed after 6 days. The level of fluorescence in the population was measured by counting 5 × 10^3 ^cells per timepoint. The filled area indicates the uninduced cells, while the green line represents the induced population. The Y axis indicates the number of cells counted, whilst the X axis shows the level of fluorescence in AFU.

In an attempt to produce a more homogeneous induction profile, we constructed a derivative of pLEW13 in which the T7 RNA polymerase and *tetR *genes were transcribed from the *T. cruzi *rRNA promoter (Methods). Cells were transformed with this plasmid (pTcrRNA-T7tet) and selected at 100 μg ml^-1 ^G418. The transformants were resistant to 2 mg ml^-1 ^G418, with no lag phase, indicative that the rRNA promoter was driving high level expression of the *neo *gene. These cells were then electroporated with the inducible vector pTcINDEX-RFP. Parasites were cloned immediately after electroporation. FACS analysis of several independent clones confirmed that expression of RFP was tetracycline-regulated, but again the response was heterogeneous within clonal populations (Fig. 7).

As these clones showed a somewhat higher background level of RFP expression (especially clone CL [pTcrRNA-T7Tet]:RFP C6), we tested the luciferase construct in this background. 5 clones were generated in the CL [pLEW13] line and 5 in the CL [pTcrRNA-T7Tet] background. Each cell line was induced for 48 hours with 250 ng ml^-1 ^tetracycline, and then assayed for luciferase activity. The results (Table 3) indicated inducible luciferase activity in all cell lines tested. However, there was a much lower level of leakiness in the CL [pLEW13] cell line than the CL [pTcrRNA-T7Tet] cell line. Whilst the former exhibited a 10–30 fold increase in luciferase activity, the latter displayed only a 5 to 9-fold increase. A similar effect was noted in the inducible system created for *Leishmania *[18]. Consequently, for applications in which a tightly regulated system is required, the CL [pLEW13] cell line appears to be much more suitable.

### Expression and localisation of epitope-tagged superoxide dismutase

To assign a biological role to a protein it is necessary to know its subcellular location. Generation of specific antibodies against every protein of interest is costly, time-consuming and not always successful. We therefore made a derivative of pTcINDEX with a c-myc epitope tag inserted next to the polylinker to facilitate localisation of induced proteins (pTcINDEX-C-myc, Fig. 2B). This could also provide a simple method to follow the induction by western blotting. To test the vector, we chose the *T. cruzi *superoxide dismutase A gene (*TcSOD A*), which encodes an isoform with a predicted mitochondrial targeting sequence. The *T. brucei *orthologue of this gene is targeted to the mitochondrion [27,28].

**Figure 8:**
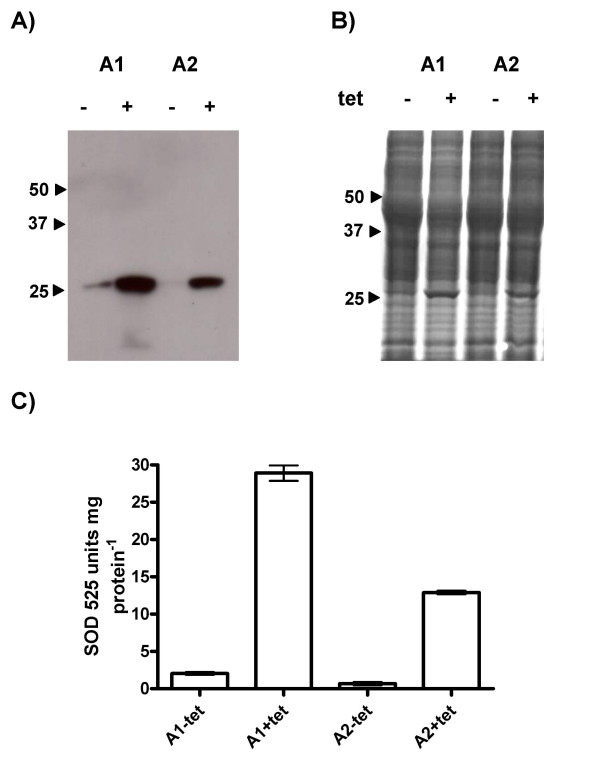
**Inducible expression of TcSOD A**. Two clones containing pTcINDEX-SOD A9E10 on a CL-Brener [pLEW13] background were induced with 0.25 μg ml^-1 ^tetracycline for five days. Replicate cultures were grown in the absence of tetracycline. Protein extracts were made and analysed by SDS-PAGE, western blotting and enzyme assay. A) Western blot of a gel stained with the mouse monoclonal 9E10. A single band of approximately 25 kDa was recognised by the antibody. B) Coomassie stained SDS-PAGE gel showing lysates from control and induced populations of clones A1 and A2. Note the intense band appearing in the induced lanes, at the position of the band recognised by the antibody in A. C) Relative SOD activities of the trypanosome lysates as measured using the SOD 525 assay system. Each assay was performed in triplicate. Clone A1 showed a 14:1 ratio of SOD activity between induced and uninduced cells, while clone A2 showed an 18:1 ratio.

The *TcSOD A *gene was inserted into pTcINDEX-C-myc such that the epitope tag was located at the carboxyl-terminus of the fusion protein (Fig. 2B). CL-Brener [pLEW13] epimastigotes were transformed as previously. Two clones were characterised (A1 and A2). With both, an induced band was visible after western blotting (Fig. 8A). In the induced cells, the corresponding bands were visible on a Coomassie stained gel (Fig. 8B). The upregulated SOD was enzymatically active (Fig. 8C), with induced cells showing a 14- and 18-fold increase over the control lines, respectively. This represents the total cellular SOD activity. Since there are four distinct isoforms in trypanosomatids [27,28], it is clear that the level of SOD A overexpression considerably exceeds 18-fold.

**Figure 9:**
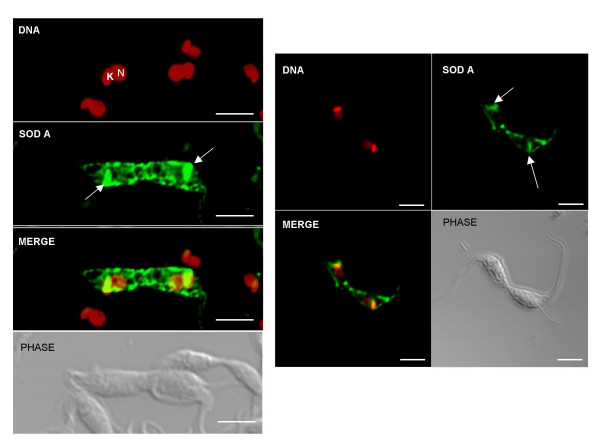
**Immunofluorescence localisation of epitope tagged TcSOD A**. Cells were induced as in Fig [8]. The parasites were fixed in paraformaldehyde and stained with mouse monoclonal anti-c-myc 9E10. Slides were examined on a Zeiss LSM 510 confocal microscope. The epitope tagged protein is shown as green fluorescence with the DNA stained red. Arrows indicate the strong staining of a structure adjacent to the kinetoplast (K). The nucleus is indicated (N). The white bar indicates 5 μm. The phase image is shown for comparison. Both images show cells in the process of dividing.

It was important to confirm that the SOD A was targeted correctly since overexpression might lead to mis-targeting or blocking of the trafficking pathway. Cells were stained with an antibody against the carboxyl-terminal epitope tag and examined by microscopy (Fig. 9). The immunofluorescence showed targeting of the induced protein to the single lattice-like mitochondrion of the trypanosome with a concentration in a rod-like structure next to, or on top of, the kinetoplast (mitochondrial) DNA. The exact nature of this structure is unclear, but the consistent proximity to the kinetoplast suggests a possible role in protection of the replicating kDNA from reactive oxygen species.

## Discussion

We have constructed a stable tetracycline-regulated expression vector for *T. cruzi *and tested several of the associated features using two marker genes, luciferase and *RFP*, and an endogenous gene *TcSOD A*. These experiments demonstrate that the system should be sufficiently robust to have widespread application in the functional analysis of parasite genes. Initially, we produced stable cell lines that constitutively expressed T7 RNA polymerase and the tetR protein using a vector system (pLEW13) originally constructed for the African trypanosome. We were able to confirm constitutive expression of both genes in transformed cells, even though the input plasmid completely lacked *T. cruzi *sequences. This type of phenomenon has previously been observed in *Leishmania *[21,29], but not in *T. cruzi *. Addition of the spliced leader sequence to each transcript occurred at the same sites as used in *T. brucei *(Fig. 2D). Analysis of the transformed cells indicated that the pLEW13 was propagated as an episome made up of multiple head-to-tail copies of the vector. This organisation has commonly been observed for episomal constructs in both *Leishmania *and *T. cruzi *[21] and is thought to arise by insertional duplication. We used a multicopy episome, rather than single integrated copies of the T7 RNA polymerase and *tetR *genes, to decrease the possibility of selection for mutations which could rescue dominant-negative or conditional knockout cell lines. Such rescue mutants occur readily in the *T. brucei *system which relies on single copies of each gene [30,31]. Episomes have been shown to be maintained in the absence of selection for up to six months, and during passage through mammalian cells and insect vectors in *T. cruzi *[21,32].

The inducible expression vector (pTcINDEX) was designed to integrate into the transcriptionally silent ribosomal RNA spacer region. We judged this to be important for two reasons. Firstly, so that in its repressed state, with the tetR protein bound tightly to the tetO, the integrated expression cassette did not block the transcription of downstream genes, and secondly, so that run-through pol II transcription occurring from upstream genes did not interfere with repression. In trypanosomatids, linked protein coding genes are organised into large polycistronic transcription units and transcriptional termination has not been fully characterised for any RNA polymerase. The pTcINDEX vector was also designed so that the *hyg *drug selectable marker was under the control of a constitutive T7 promoter. Thus expression of T7 RNA polymerase is necessary for cells to display resistance to hygromycin and continued selection with G418 is no longer required to maintain the presence of the pLEW13 episome.

In the pLEW13 transformed cells, our experiments indicate that the level of inducible expression may vary from gene to gene, but that any background, due to insufficiently tight repression of promoter activity, is likely to be low. With pTcINDEX-RFP transformed cells, we were unable to detect any fluorescence by microscopy, and only a low level by FACS, in the absence of tetracycline (0.2% – 3% of cells counted depending on the clone, Fig. 6 filled area, Table 2). Similarly, with pTcINDEX-Luc transformed cells, detection of the luciferase transcript on northern blots was tetracycline-dependent (Fig. 4A). However, there was a reproducibly detectable level of enzyme activity associated with non-induced cells. This background appeared relatively constant in a given clone, although it did vary between clones. We also noticed that the level to which luciferase activity was induced (up to 60-fold) was low compared to that of the mRNA (>100-fold), suggesting that expression may be regulated at the level of translation or that the luciferase protein may be less stable in trypanosomes.

Both microscopy and FACS analysis showed that there was a wide variation in the level of inducible expression of RFP in individual cells, even within a cloned population, ranging from <10- to >1000-fold. When the machinery required for inducible expression (the T7 RNA polymerase and *tetR *genes) was placed under the control of an rRNA promoter, the induction profile did not become more homogeneous, but the background expression increased significantly. Thus, no advantage was gained and repression was decreased using this construct. This could be due to high level expression of tetR resulting in aggregation and loss of function as has been postulated in the *Leishmania *system [18].

Cell-by-cell analysis in the manner described here, has not, to our knowledge, been performed with the *T. brucei *or *Leishmania *inducible expression systems, although a heterogeneous pattern of induction has been observed using a tetracycline-regulated promoter to drive expression of GFP in yeast [33]. This type of variation is a common feature of eukaryotic cells and is thought to reflect the inherently stochastic nature of gene expression at the level of both transcription and translation [34-36]. Recent work from several laboratories has shown that stochastic elements play a significant part in generating "noise" in gene expression, i.e. the variation in expression of a given protein between genetically identical individuals in a population under the same conditions [37-41]. Thus, the pattern of RFP fluorescence observed using FACS analysis and microscopy may be regarded as a "snapshot" of the fluctuating levels of RFP expression that occur even within a clonal population. Indeed, a recent report [42] has shown that even when a marker gene (GFP) is integrated into the *T. cruzi *genome under the control of a strong constitutive rRNA promoter, the FACS profile of a stably transformed cell line is remarkably heterogeneous, with approximately 25–30% of cells expressing little or no detectable GFP at a given time. This suggests that variation in protein levels between individual cells may be an inherent feature of *T. cruzi *gene expression, rather than a consequence of episomal expression of the polymerase and repressor genes. It is notable that the *T. cruzi *genome contains many highly polymorphic multigene families encoding surface proteins which are co-expressed in a given population. Therefore, stochastic expression of surface antigens between individual cells of a population may be an important immune evasion strategy [43]. It has been hypothesised that micro-organisms benefit from noise in gene expression as this allows a population to respond more rapidly to changes in their environment and decreases the chances of cells becoming mired in inappropriate epigenetic states [44].

The availability of our inducible expression system will provide new approaches for the functional analysis of genes in *T. cruzi*. It will allow the study of proteins that may be toxic if constitutively expressed, enable the generation of conditional knockouts of essential genes and facilitate functional knockouts by means of overexpression of dominant-negative protein mutants. The level of overexpression achieved with SOD A (Fig. 8) suggests that a dominant-negative approach will be feasible, since in such a system the mutated protein must be expressed at significantly higher levels than the endogenous enzyme.

Modulation of expression levels by changing the concentration of tetracycline could also be important for conditional knockout experiments. This will enable the transfected gene to be expressed at a similar level to the endogenous copy, thereby preventing unforeseen phenotypic consequences due to overexpression. An advantage of using tetracycline as the inducer is that the expression system can be applied to the study of enzyme function throughout the life-cycle. For example, it should be possible to investigate the development of transformed parasites within tissue-culture cells using the tetracycline analogue doxycycline, which has been used to regulate murine gene expression in transgenic (Tet-On) mice [45]. The combination of episomally expressed T7 RNA polymerase and tetR with an inducible vector which can integrate into the rRNA locus in both group I and group II parasites also means that this system is transferable to any strain of *T. cruzi*. pTcINDEX and pTcINDEX-C-myc are freely available to members of the trypanosomatid research community.

## Conclusion

We have designed and tested a user-friendly tetracycline-regulatable expression vector, pTcINDEX, for the protozoan parasite *T. cruzi*. This vector has been used to generate cell lines bearing inducible copies of luciferase, RFP and SOD A. The levels of repression and induction achieved lead us to believe that this vector will be useful for creating both conditional knockouts and dominant-negative mutants of *T. cruzi*, an organism for which RNAi based approaches are not applicable.

## Methods

### Parasite maintenance and genetic manipulation

Epimastigotes of *T. cruzi *CL-Brener were maintained at 27°C in RPMI-1640 medium as described previously [46], except that we used 5% tetracycline-free fetal calf serum (Autogen Bioclear). Parasites were transformed by electroporation using a Bio-Rad Gene Pulser II, placed into fresh medium and incubated for 48 hours to allow expression of the drug-selectable marker. The appropriate drug was then added (G418 at 100–200 μg ml^-1 ^or hygromycin at 100 μg ml^-1^) and the cells incubated for a further four to six weeks to allow selection of transformants. For direct cloning, parasites were resuspended in 24 ml of fresh medium directly after electroporation. 1 ml was then transferred to each well of a 24-well plate and the cells allowed to grow for 48 hours prior to addition of the selective drug. Typically, between 2 and 5 clones were generated per 24-well plate.

### Plasmid construction

The inducible expression vector pTcINDEX (Fig. 2) was based on the *T. brucei *RNAi vector pZJM [8](a gift from Paul Englund). First, the inverted promoter fragment of pZJM, which contains bi-directional T7 promoters, was isolated by digestion with *Kpn *I and *Bam *HI. This fragment was then subcloned into pGEM3zf+ (Promega) to produce vector pGEMT7Tet2. In parallel, a *hyg *gene flanked by the processing signals from the *T. cruzi *glycosomal glyceraldehdye-3-phosphate dehydrogenase gene[21,46] was inserted into the *Eco *RV site of pBluescript KS(-). A constitutive T7 promoter was then inserted upstream of the *hyg *gene after generation of the appropriate fragment by PCR. This 2.4 kb cassette was isolated following *Kpn *I and *Sac *I digestion and sub-cloned into pGEMT7Tet2, upstream of the fragment derived from pZJM, to create pGEMhygT7-3. Since the pGEM backbone contains an additional unwanted T7 promoter, the whole insert fragment was liberated by *Bam *HI/*Sac *I digestion and inserted into pUC19ΔH (pUC19 with the *Hin*d III site deleted by end-filling).

The ribosomal RNA non-transcribed spacer and promoter region were amplified from genomic DNA of *T. cruzi *in two pieces of 1.8 kb and 0.3 kb to allow the introduction of a unique *Spe *I site for vector linearization prior to transfection. These pieces were generated using the primer pairs:

5'-TTTACTAGTAGCTCGGTGCACCCTG, 5'-GGGGAGCTCACACAAATGGACGGTTA and, 5'-GGGGAGCTCATTTGTGTCTAGTACATC, 5'-GGGACTAGTCTGAGGCATGCATGGCTA.

The fragments were ligated together then cloned into the *Sac *I site immediately downstream of the *hyg *cassette (as shown in Fig. 2) to produce plasmid pTcIRi. To construct the inducible expression vector, we modified pTcIRi by removing the antisense promoter and adding a polylinker and RNA processing signals. Briefly, pTcIRi was partially digested with *Sac *I and *Hin*d III. The *Sac *I/*Hin*d III fragment containing the *hyg *gene and the sense strand inducible promoter was cloned into pGEM3zf+ (Promega) to create pGEMTcI. The T7 transcriptional terminator from pTcIRi was amplified with primers 5'-TTTCTCGAGCGGCCGCCGGCATCGATACGCGTGGATCCTCGCGAATCAGGTGCTAGCCCGCT and 5'-TTTAAGCTTGATCCCCGGATATAGTT. This fragment, which also incorporated a multiple cloning site (underlined, Fig. 2A), was inserted into *Xho *I/*Hin*d III digested pGEMTcI.

The splice acceptor site from the ribosomal protein P2β locus was amplified from pTREX-n [25] (a gift from Mariano Levin), using primers which added an *Xho *I site to the 5' end and a *Not *I site to the 3'end. This 212 bp fragment was cloned into the corresponding sites of the polylinker. The actin intergenic sequence [Acc. No. U20234], which contains a putative polyadenylation signal, was amplified from genomic DNA of *T. cruzi *using primers: 5'-CCCGGATCGTCGCGAGGCAGGCCCAAGCA and 5'-CCCGATATCGTCAGACATCCTTAGAA. The resulting 424 bp fragment was then digested with *Bam *HI and *Eco *RV and cloned into the *Bam *HI and *Nru *I sites of the polylinker. The entire insert was again transferred to pUC19 via a partial *Sac *I/*Hin*d III digest to produce the final expression construct pTcINDEX (Fig. 2).

The luciferase coding sequence was obtained from pGEM-luc (Promega). The plasmid was digested with *Sal *I and end-filled by the Klenow fragment. The gene was then excised by digestion with *Bam *HI. pTcINDEX was digested with *Bam *HI and *Nru *I and the luciferase gene was ligated into the vector to produce pTcINDEX-Luc. To obtain the gene encoding the red fluorescent protein (RFP), construct pTEX-Red [47] was first digested with *Bam *HI and *Bgl *II and re-ligated to delete awkward restriction sites. The modified plasmid was then cut with *Spe *I and the ends filled by Klenow treatment. The RFP gene was liberated by digesting the linear plasmid with *Cla *I and cloned into *Nae *I/*Cla *I sites of pTcINDEX to create pTcINDEX-RFP.

pLEW13 was modified to include a *T. cruzi *rRNA promoter to drive expression of the T7 RNA polymerase, *neo *and *tetR *genes. Briefly the *Sac *I fragment carrying the rRNA promoter and upstream spacer region was removed from pTcINDEX and subcloned into pUC19 to make pUC-TcrRNA. The T7 RNA polymerase, *neo *and *TetR *genes were removed from pLEW13 on a 5.9 kb fragment using *Eco*RV and *Stu *I. This fragment was then cloned into the unique *Spe *I site in the rRNA promoter fragment of pUC-TcrRNA, such that the transcription initiation point was upstream of the T7 polymerase gene. This derivative of pLEW13 was named pTcrRNA-T7Tet.

We created an epitope tagging vector by cloning the BPP1-myc fusion gene from pTEX-BPP1-9E10 into *Bam *HI/*Nru *I digested pTcINDEX [48]. This fusion gene contains a unique *Eco *RV site between the *BPP1 *ORF and the c-myc tag such that any gene of interest can replace the *BPP1 *coding sequence and be cloned in-frame with the tag (Fig. 2B). The epitope tag encodes the sequence EQKLISEEDL*, where * indicates a translational stop. This vector was named pTcINDEX-C-myc where the uppercase C denotes that the tag is fused to the carboxyl terminal of the protein of interest. To make an inducible tagged copy of TcSOD A, the gene (>Tc00.1047053509775.40 [49]) was amplified from genomic DNA of the CL-Brener strain using the following primers:

SOD A F: gggggatccATGTTGAGACGTGCGGTGAA

SOD A R: ggttgatatcTTTTATTGCCTGCGCAT

where underlining indicates restriction sites introduced for ease of cloning. The 699 bp product was digested with *Bam *HI and *Eco *RV and ligated into *Bam *HI/*Eco *RV digested pTcINDEX-C-myc, such that the *SOD *A ORF was in-frame with the carboxyl terminal epitope tag under the control of the inducible T7 promoter. The construct was confirmed by DNA sequencing.

### Nucleic acid analysis

DNA and RNA were prepared and purified using Qiagen kits as per manufacturer's instructions. RNA was quantified using a 2100 Bioanalyzer with RNA 6000 Nano Labchip (Agilent). Southern and northern blotting were carried out using standard protocols. Reverse transcriptase PCR (RT-PCR) was carried out using the Access RT-PCR kit (Promega) and primers:

Spliced Leader sense 5'-GGGGGATCCACAGTTTCTGTACTATATTG

T7 Polymerase antisense 5'-TCGTAAGACTCATGCTCAA

*Neo *antisense 5'-CCTCGTCCTGACAGTTCAT

*tetR *antisense 5'-TGCCTATCTAACATCTCA

The products were cloned into pGEM-T (Promega) and sequenced using a dye terminator cycle-sequencing kit (Applied Biosystems) and an ABI Prism 377 DNA sequencer. For CHEFE analysis parasite blocks were made as described [50]. The chromosomes were resolved on a CHEFmapper system (Bio-Rad) using the auto-algorithm and conditions as detailed in figure legends.

### Induction of gene expression

For induction experiments, epimastigotes were cultured in 25 cm^3 ^flasks at 27°C and maintained in logarithmic growth phase (10^6 ^– 10^7 ^cells ml^-1^). Control cells were grown in tetracycline-free medium, whilst the induced cells were cultured in medium supplemented with the stated concentration of tetracycline. We found that the doubling time of wild type parasites was unaffected by low concentrations of tetracycline, but was increased by 13% at 5 μg ml^-1 ^and by 30% at 10 μg ml^-1^. Inductions were carried out over variable time courses as stated in figure legends.

### Luciferase assays

Epimastigotes transformed with pTcINDEX-Luc were grown as described above. At each time point an aliquot was removed, pelleted and washed in PBS (137 mM NaCl, 4 mM Na_2_HPO_4_, 1.7 mM KH_2_PO_4_, 2.7 mM KCl). Cell pellets were frozen in liquid nitrogen and stored at -80°C. For the luciferase assay, the pellet was resuspended in 500 μl of cell culture lysis reagent (Promega). Lysates were vortexed for 15 seconds and the debris removed by centrifugation. Activity was measured using the luciferase assay system (Promega) and light emission measured on a β-plate counter (Wallac). The linear detection limits of the counter were measured using serial dilutions of Quanti-Lum recombinant luciferase (Promega). Protein concentrations were determined by the BCA assay (Pierce) using equivalent amounts of cells lysed in PBS, as the lysis reagent is incompatible with the protein assay.

### Fluorescence microscopy and FACS analysis of RFP expression

RFP expression was examined by confocal microscopy on a Zeiss LSM 510 Axioplan microscope. Transformed parasites were induced as described above. At each time point, an aliquot of cells (10^7^) was removed, pelleted, washed in PBS and then fixed for 30 minutes in 4% paraformaldehyde/PBS. Cells were then washed and resuspended in 5 ml PBS. 20 μl of the suspension was dotted onto a single well of a 12-well slide. DNA was stained by adding 50 nM TOTO-3 (Molecular Probes) in 10 mg ml^-1 ^RNAse A/0.1% saponin/PBS to each well, incubating at room temperature for 20 minutes, then washing twice in PBS. Slides were mounted in 1:1 PBS/glycerol. For FACS analysis, cells were fixed as above and finally resuspended at 10^7 ^parasites ml^-1^. 5 × 10^3 ^– 10^4 ^cells per time point were counted on a FacScan or FacsCalibur (Becton Dickinson). Data were analysed using Cellquest™ software (BD Sciences).

### Protein extraction and analysis

For western blot and SOD activity assays, cells were pelleted, and washed once in PBS. The cells were pelleted again and resuspended in lysis buffer (PBS supplemented with proteinase inhibitors, Roche). The cell suspension was freeze-thawed three times in liquid nitrogen then sonicated. Membrane debris was removed by centrifugation (10,000 g for 10 mins). The supernatant was removed to a sterile tube and stored at -80°C. SDS-PAGE and western blotting were carried out as per standard protocols. The western blots were probed with mouse monoclonal c-Myc (9E10) (cat no. *sc-40*, Santa Cruz Biotechnology Inc.) diluted 1:2000. For SOD activity assays the Bioxytech™ SOD 525 (Oxis Research) kit was used as per manufacturer's instructions.

### Immunofluorescence

To check the localisation of the tagged SOD A, epimastigotes were fixed in 4% paraformaldehyde and dried onto slides. The slides were stained with mouse monoclonal c-Myc (9E10) (diluted 1:200) and then Alexafluor 488 conjugated goat anti-mouse (diluted 1:400 Molecular Probes). DNA was stained with DAPI. Slides were examined on a Zeiss LSM 510 confocal laser scanning microscope.

## Authors' contributions

MCT designed the vectors and all derivatives thereof except where stated, and carried out all practical work involved in this study. JMK participated in the conception and design of the study and helped to draft the manuscript. Both authors read and approved the final manuscript.

## Figures and Tables

**Table 1:** Inducible luciferase activity in independent clones transformed with pTcINDEX-Luc

**Cell line**	**Uninduced**	**0.5 μg ml**^**-1 **^**tet**	**Ratio +tet/-tet**
CL-Luc 1	4988 (234)	185275 (16417)	37.1
CL-Luc 2	2321 (167)	24105 (1539)	10.4
CL-Luc 3	2497 (54)	34987 (2859)	14.0
CL- Luc 4	712 (39)	3151 (464)	4.4
CL-Luc 5	4066 (386)	8333 (532)	2.0

Each clone was induced with 0.5 μg ml^-1 ^tetracycline. After 24 hours, extracts were tested for luciferase activity (Methods). Each extract was assayed in triplicate. Controls were identical cultures maintained in the absence of tetracycline. The activity is represented as mean relative light units per 5 × 10^4 ^epimastigotes. Figures in parentheses represent standard error of the mean.

**Table 2:** Inducible RFP expression in independent clones transformed with pTcINDEX-RFP.

**Cell line**	**Uninduced**	**0.5 μg ml**^**-1 **^**tet**	**Ratio +tet/-tet**
CL-RFP C2	0.3	71.8	239
CL-RFP A2	3.3	31.7	9.6
CL-RFP D6	0.7	25.8	36.8
CL-RFP D2	0.3	12.8	42.7
CL- RFP C5	1.1	17.5	15.9
CL-RFP B1	0.6	2.0	3.3

Each clone was induced with 0.5 μg ml^-1 ^tetracycline. After 5 days, cells were fixed and analysed on a FacsCalibur. Controls were identical cultures maintained in the absence of tetracycline. The data are presented as percentage of cells registering greater than 6 arbitrary fluorescence units (AFU).

**Table 3:** Inducible luciferase activity in independent clones transformed with pTcINDEX-Luc in pLEW13 or pTcrRNA-T7Tet background.

**Cell line**	**Uninduced**	**0.25 μg ml**^**-1 **^**tet**	**Ratio +tet/-tet**
CL-LA1N (pLEW13)	338 (16)	9831 (411)	29
CL-L1D4 (pLEW13)	3016 (36)	75792 (4195)	25
CL-LB5N(pLEW13)	393 (7)	7426 (281)	19
CL-L4B1(pLEW13)	303 (5)	3196 (256)	11
CL-L4D5(pLEW13)	848 (37)	18003 (613)	21
			
CL-L1B6 (pTcrRNA-T7Tet)	83249 (2920)	412729 (33287)	5
CL-L1C4 (pTcrRNA-T7Tet)	76625 (4521)	709201 (12746)	9
CL-L2A4 (pTcrRNA-T7Tet)	91785 (4306)	425708 (15432)	5
CL-L2D4 (pTcrRNA-T7Tet)	92374 (7511)	769333 (44171)	8
CL-L1A6 (pTcrRNA-T7Tet)	86922 (2736)	316358 (16381)	3

Each clone was induced with 0.25 μg ml^-1 ^tetracycline. After 48 hours, extracts were tested for luciferase activity (Methods). Each extract was assayed in triplicate. Controls were identical cultures maintained in the absence of tetracycline. The activity is represented as mean relative light units per 5 × 10^4 ^epimastigotes. Figures in parentheses represent standard error of the mean.
